# Supplementary material for: Cortical bone maturation in mice requires SOCS3 suppression of gp130/STAT3 signalling in osteocytes
Source: eLife. 2020 May 27;9:e56666. doi: 10.7554/eLife.56666 (PMC7253175; doi:10.7554/eLife.56666)

Source data: Figure 4

Uncropped gels for phospho-STAT3 (left panels) and total STAT3 / pan-actin (right panels) for calvarial samples from *Socs3*$^{f/f}$ and *Dmp1*^{Cre}:*Socs3*$^{f/f}$ mice treated with OSM (A), LIF (B) or IL-11 (C). Details of molecular weight markers and legends for the gels are in Figure 4.

A

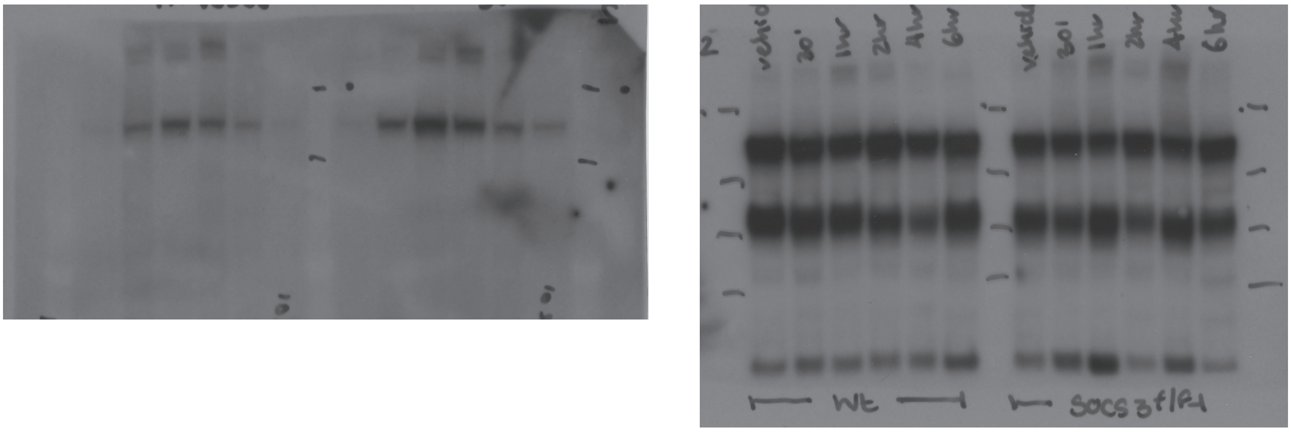

B

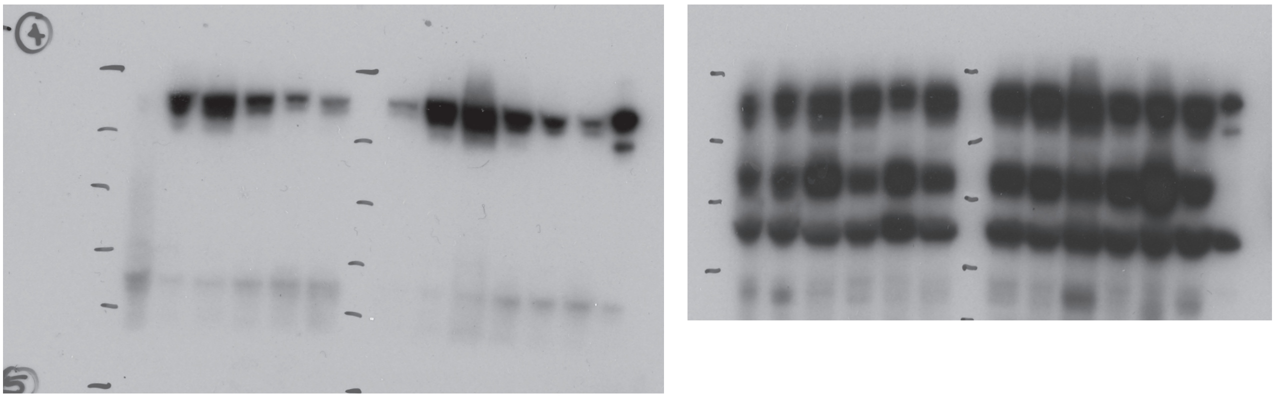

C

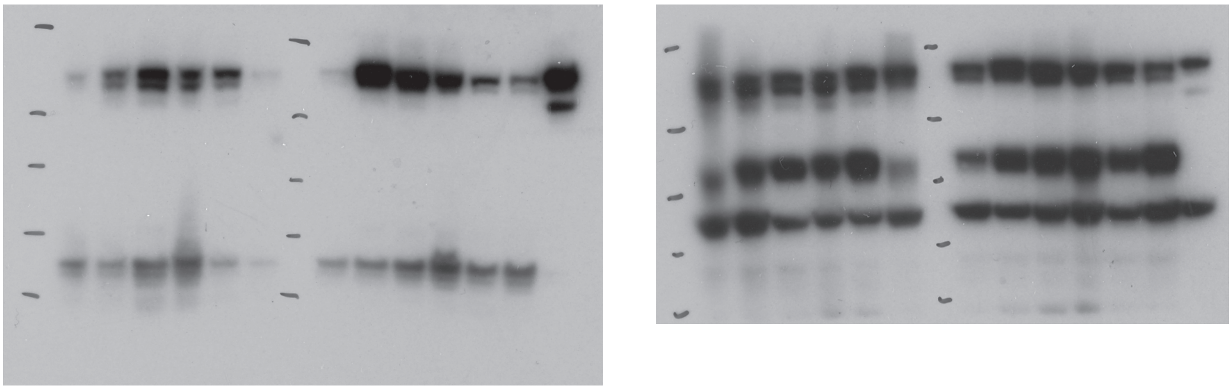

Supplement: Figure 4—source data 1. — Details of molecular weight markers and legends for the gels are in Figure 4. [file elife-56666-fig4-data1.pdf]
